# Collateral responses to classical cytotoxic chemotherapies are heterogeneous and sensitivities are sparse

**DOI:** 10.1038/s41598-022-09319-1

**Published:** 2022-03-31

**Authors:** Simona Dalin, Beatrice Grauman-Boss, Douglas A. Lauffenburger, Michael T. Hemann

**Affiliations:** 1 Department of Biology, Massachusetts Institute of Technology, Cambridge, MA USA; 2 Koch Institute for Integrative Cancer Research, Massachusetts Institute of Technology, Cambridge, MA USA; 3 Department of Biological Engineering, Massachusetts Institute of Technology, Room: 16-343, Cambridge, MA 02139 USA

**Keywords:** Cancer therapeutic resistance, Chemotherapy, Tumour heterogeneity

## Abstract

Chemotherapy resistance is a major obstacle to curing cancer patients. Combination drug regimens have shown promise as a method to overcome resistance; however, to date only some cancers have been cured with this method. Collateral sensitivity—the phenomenon whereby resistance to one drug is co-occurrent with sensitivity to a second drug—has been gaining traction as a promising new concept to guide rational design of combination regimens. Here we evolved over 100 subclones of the Eµ-Myc; p19^ARF−/−^ cell line to be resistant to one of four classical chemotherapy agents: doxorubicin, vincristine, paclitaxel, and cisplatin. We then surveyed collateral responses to acquisition of resistance to these agents. Although numerous collateral sensitivities have been documented for antibiotics and targeted cancer therapies, we observed only one collateral sensitivity: half of cell lines that acquired resistance to paclitaxel also acquired a collateral sensitivity to verapamil. However, we found that the mechanism of this collateral sensitivity was unrelated to the mechanism of paclitaxel resistance. Interestingly, we observed heterogeneity in the phenotypic response to acquisition of resistance to most of the drugs we tested, most notably for paclitaxel, suggesting the existence of multiple different states of resistance. Surprisingly, this phenotypic heterogeneity in paclitaxel resistant cell lines was unrelated to transcriptomic heterogeneity among those cell lines. These features of phenotypic and transcriptomic heterogeneity must be taken into account in future studies of treated tumor subclones and in design of chemotherapy combinations.

## Introduction

Since the 1950s, clinicians have combated intrinsic chemotherapy resistance by treating patients with combinations of chemotherapies that have non-overlapping toxicities thereby allowing for higher doses. However, this method has resulted in an overall 5-year survival rate across all cancers of only 68%^1^. The antibiotic resistance field is facing a similar problem—rates of antibiotic resistance doubled between 2002 and 2014, and there are 23,000 deaths in the US each year due to antibiotic-resistant infections, according to the Centers for Disease Control and Prevention^2^. In response, researchers in the antibiotic resistance field have studied cases where resistance to one antibiotic causes, or is co-occurrent with, sensitivity to a second antibiotic—termed collateral sensitivity^3^. In theory, using collaterally sensitive drug pairs in combination or sequentially could select against the emergence of resistant microbes and thus reduce or prevent the emergence of antibiotic resistance^4^.

Recently, the concept of collateral sensitivity has begun to be explored in the context of cancer chemotherapy as well. For example, in 2016, Zhao et al. found that in Philadelphia chromosome-positive acute lymphoblastic leukemia (ALL), resistance to dasatinib, a BCR-ABL1 inhibitor, can cause collateral sensitivity to crizotinib, foretinib, vandetanib, and cabozantinib—cMET and/or VEGFR inhibitors^5^. The following year, Andrew Dhawan and colleagues characterized collateral sensitivities to several tyrosine kinase inhibitors (TKIs) in ALK-positive non-small cell lung cancer (NSCLC). They found that cell lines resistant to first-line TKIs are often sensitized to non-TKIs including the classical chemotherapies etoposide and pemetrexed^6^. In 2020 a study from the same group showed that resistance to combinations of classical chemotherapies in Ewing’s sarcoma causes resistance to the drugs in the combinations, as well as to actinomycin D, and potentially sensitivity to SP-2509, a lysine-specific demethylase 1 inhibitor^7^. Recently, Mueller et al. found that resistance to PRMT5 inhibition causes collateral sensitivity to paclitaxel due to upregulation of *Stmn2*^8^.

While the growing knowledge of collateral sensitivities to targeted therapies is encouraging, most patients today are treated with at least one classical chemotherapy. A close review of the literature reveals several examples of likely collateral sensitivities between classical therapies. Vinblastine and paclitaxel have opposing mechanisms of action—the former destabilizes microtubules, and the latter stabilizes microtubules. They also have opposing mechanisms of resistance, vinblastine resistance can result from stabilizing mutations in α- and β-tubulin, while paclitaxel resistance can result from destabilizing mutations in α- and β-tubulin. When these resistance mechanisms are present, vinblastine-resistant cell lines are sensitive to paclitaxel, and vice versa^9^. Additionally, there is experimental and clinical data suggesting that, at least in some cases, cisplatin resistance can cause sensitivity to paclitaxel, and vice versa^10–12^. The mechanism behind this effect remains unclear, however, this combination has been found to be effective to treat patients with ovarian, breast, lung, skin, and head and neck tumors^13^.

While there are individual examples of collateral sensitivities to classical chemotherapies, there has not been a comprehensive survey of collateral sensitivities to single-agents in this class of drug, as has been done for antibiotics and targeted therapies. Furthermore, the dearth of known mutations that predict resistance to classical chemotherapies motivated us to define a proxy of resistant state by using the collateral effect of resistance to classical chemotherapies on sensitivity to other agents. Here, we survey collateral responses to acquired resistance to several classical therapies in the Eµ-Myc; p19^ARF−/−^ cell line which is a murine model for human Burkitt’s lymphoma^14–16^. We chose to investigate classical therapies which are commonly used in the clinic, several of which are used to treat Burkitt’s lymphoma, and several of which have collateral sensitivities described in the literature^17^. The drugs we studied are doxorubicin, a topoisomerase II poison, vincristine, a microtubule destabilizing agent, paclitaxel, a microtubule stabilizing agent, and cisplatin, a DNA crosslinker^18–20^.

Using a 96-well plate-based drug pulse and stepwise dose increase protocol, we were able to create > 15 evolutionary replicates of stable cell lines resistant to each of these four drugs. We created a protocol to measure the resulting lines’ sensitivities to a panel of 20 chemotherapies with high precision. We found only one collateral sensitivity in a subset of the cell lines we evolved. This work also revealed marked heterogeneity in collateral changes in drug sensitivity between cell lines evolved to be resistant to the same drug. Surprisingly, transcriptomic profiling of cell lines resistant to paclitaxel was unable to explain the phenotypic heterogeneity. This heterogeneity in resistance acquisition needs to be taken into account when designing classical and salvage combination regimens in order to optimize patient outcome. Similarly, potential discordance between phenotype and transcriptome must be considered when studying transcriptome as a proxy for functional biology.

## Results

### Optimized protocol to evolve chemotherapy-resistant cell lines in vitro

To study collateral effects of resistance to classical chemotherapies, we developed a protocol allowing us to evolve many cell lines to resistance to one therapy in parallel. In these experiments we used the murine Eµ-Myc; p19^ARF−/−^ cell line as a tractable model because it is chemo-sensitive, has a short doubling time of ~ 12 hours, and grows in suspension so it can be easily manipulated in small culture wells^15^.

**Figure 1:**
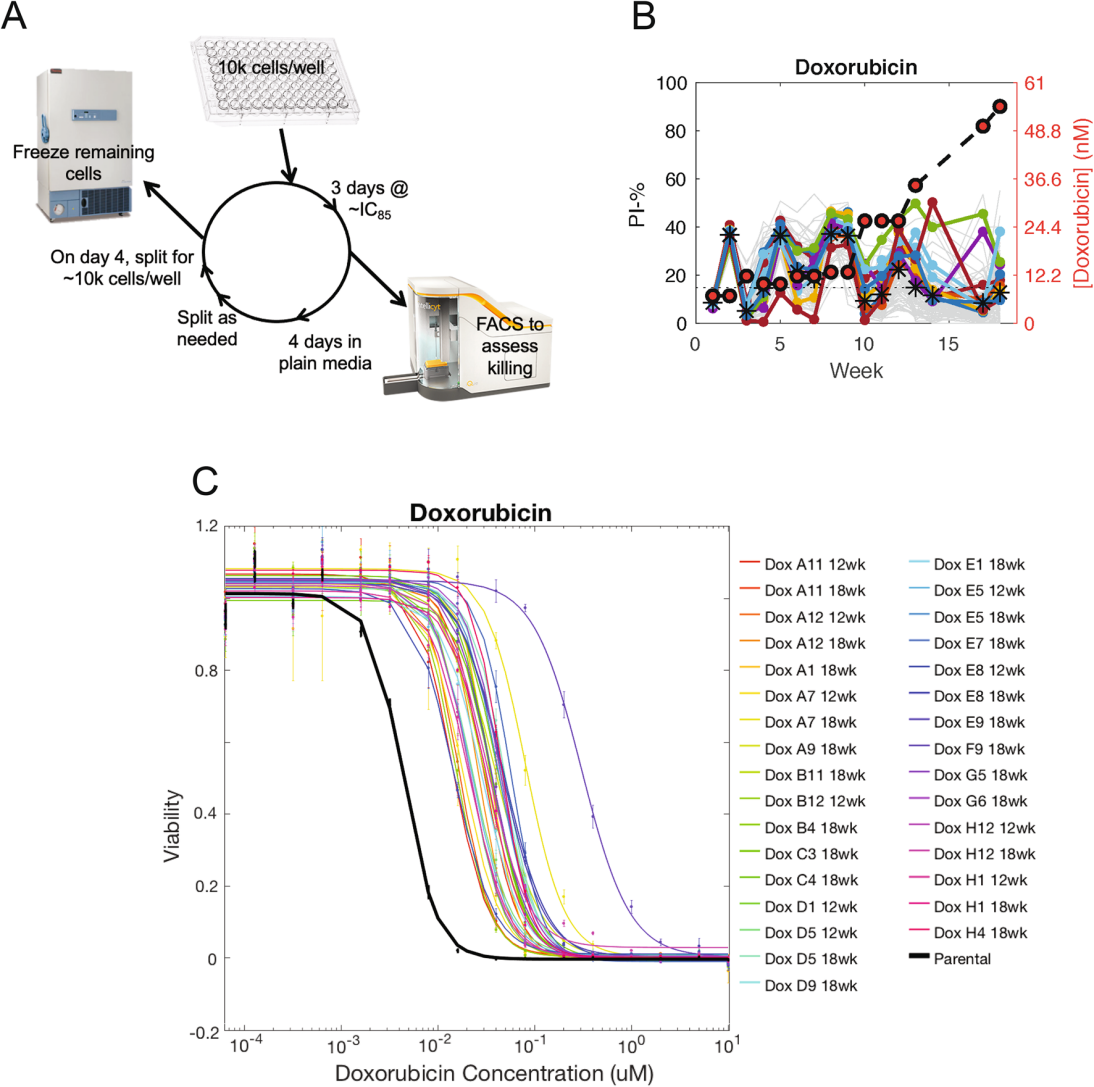
Protocol to create multiple evolutionary replicates of classical chemotherapy-resistant cell lines in parallel. **(A)** Schematic of protocol. In each cycle, 10,000 cells were seeded in each well of a 96-well plate and treated at a dose to kill 85% of cells after three days. Killing was assessed via FACS and cells were allowed to recover for four days. Cells were split, frozen, and the cycle re-started. **(B)** The fraction of live cells present in each well over each cycle of the resistance evolution experiment as assessed by PI staining (left y-axis). The dose of doxorubicin used each week is plotted with red circles on the right y-axis. Colored lines represent the most viable cell lines over the last five cycles of the experiment of the experiment. **(C)** Quantification of sensitivity to the selection drug after 12 or 18 cycles of selection.

To evolve resistance to classical chemotherapies, 10,000 cells were treated with a dose of drug expected to kill 85% of cells over the course of three days in 96-well plates. After three days, killing was assessed using PI staining via flow cytometry and drug was removed from cells. Cells were allowed to recover for four days in untreated media and wells were split as they became confluent. On day four, the plate was split to leave approximately 10,000 cells in each well which were treated again with a dose expected to kill 85% of cells based on the killing achieved the previous week. Remaining cells from the split were frozen and saved at −80 °C (Fig. 1A).

As a pilot experiment, we used this protocol to create cell lines resistant to doxorubicin. Over the course of 18 weeks, cell lines were able to grow in increasing doses of doxorubicin and, by the end of the experiment, cell lines’ EC_50_ to doxorubicin increased by 9.7 ± 0.4 fold (Fig. 1B,C).

### Screen of four doxorubicin-resistant cell lines against large drug library

**Figure 2:**
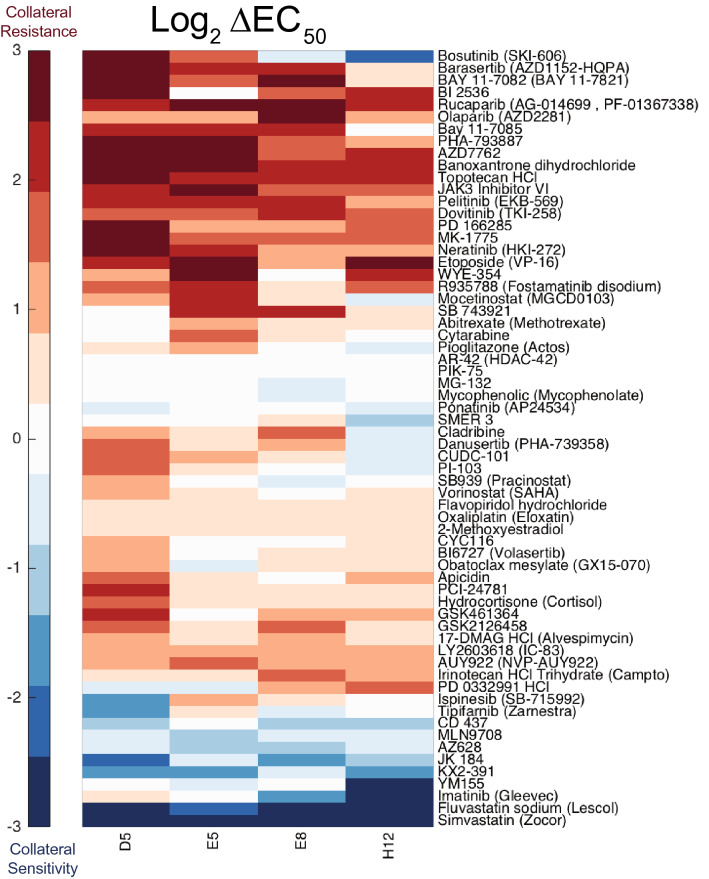
High-throughput drug screen to assess collateral responses to doxorubicin resistance. Cell lines screened are in columns, drugs with collateral effects are in rows. Colors represent the log_2_ fold change of the EC_50_ of each cell line for a drug relative to the parental cell line’s EC_50_ for that same drug. Red represents a case in which the resistant cell line also acquired resistance to a different drug—collateral resistance. Blue represents a case in which the resistant cell line acquired sensitivity to a different drug—collateral sensitivity.

To assess collateral changes in drug sensitivity, we screened four representative doxorubicin-resistant cell lines against an anti-cancer compound library containing 392 drugs. As expected, we observed collateral resistance to etoposide, another topoisomerase II inhibitor. We also observed a variety of other collateral changes in drug sensitivity, including collateral sensitivity to two HMG-CoA reductase inhibitors—fluvastatin and simvastatin (Fig. 2).

### Generated cell lines resistant to four classical chemotherapies

**Figure 3:**
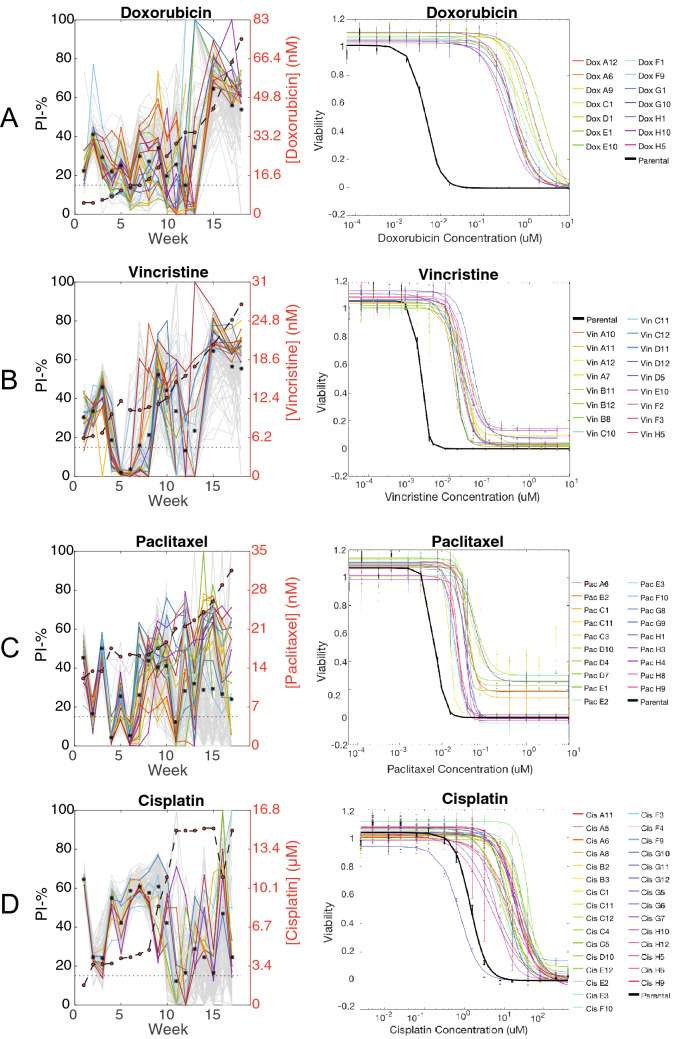
Viability of cell lines over each cycle of the resistance evolution experiment (left column) and quantification of sensitivity to the selection drug after 18 cycles (right column). In the left column, colored lines represent wells that were most viable over the last 5 weeks of the experiment. Each row represents acquired resistance to **(A)** doxorubicin, **(B)** vincristine, **(C)** paclitaxel, and **(D)** cisplatin.

We next used this method of creating resistant cell lines to generate cell lines resistant to vincristine, cisplatin, paclitaxel, as well as additional cell lines resistant to doxorubicin. We also generated control cell lines using DMSO in the place of drug. We were able to generate a total of 47 doxorubicin-resistant cell lines, 16 vincristine-resistant cell lines, 19 paclitaxel-resistant cell lines, 30 cisplatin-resistant cell lines, and 12 DMSO control cell lines. The fold change of EC_50_ to the selection drug was 206.5 ± 8.7 for doxorubicin-resistant cell lines, 10.8 ± 0.2 for vincristine-resistant cell lines, 5.2 ± 0.1 for paclitaxel-resistant cell lines, and 13.5 ± 0.3 for cisplatin-resistant cell lines (Fig. 3A–D).

### Developing a methodology to screen fewer drugs against many cell lines with high accuracy and precision

To determine collateral effects of resistance to doxorubicin, vincristine, paclitaxel, and cisplatin, we wanted to determine changes in sensitivity to chemotherapies. We developed a new dose response methodology to test a larger dose range and increase accuracy and precision of the drug sensitivity data relative to the screen we performed previously.

We reasoned that by choosing drugs that target a wide variety of biological processes, we may also gain insight into cellular processes changed over the course of acquiring resistance. For each of the chosen 20 drugs, we performed 25-fold 8-point serial dilutions in deep-well 96-well plates by hand. Each serial dilution was performed over four “quadrant plates”. We then used a Tecan liquid handler to transfer 20 μl of that serial dilution to 384-well plates. 30 μl of cells were plated on top of drug using a BioTek plate washer. After three days of incubation, we assessed viability with resazurin using a Tecan plate reader.

### Resistant cell lines exhibited extensive collateral resistance and limited collateral sensitivity

To study collateral changes in drug sensitivity resulting from classical chemotherapy resistance, we processed the raw dose response data into EC_50_ values for each drug and cell line. We then calculated the log_2_ fold-change in EC_50_ values for each cell line and drug pair relative to the parental cell line’s EC_50_ value for the same drug.

**Figure 4:**
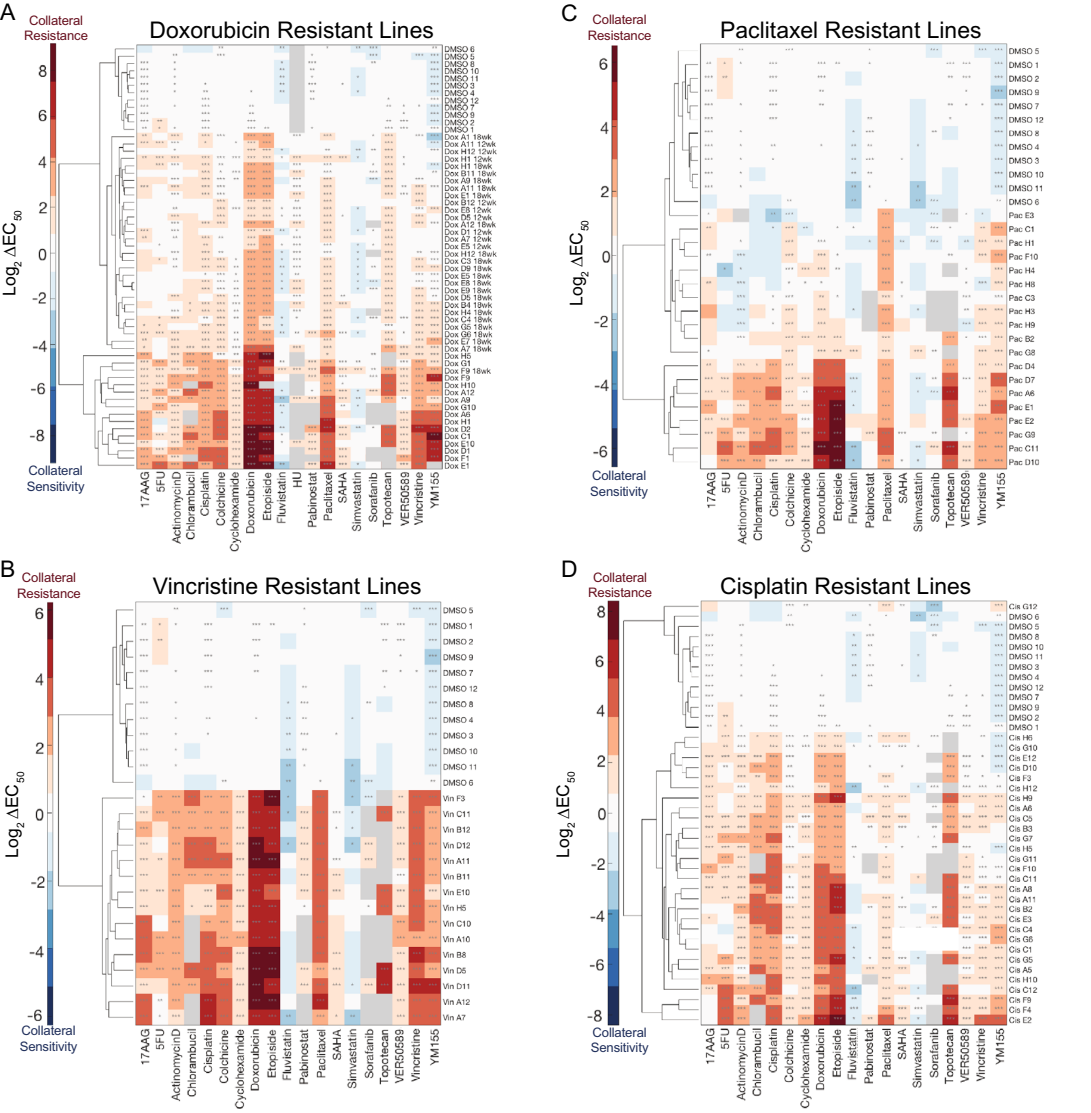
Collateral effects of acquiring resistance to **(A)** doxorubicin, **(B)** vincristine, **(C)** paclitaxel, and **(D)** cisplatin. In each plot, control cell lines cultured with DMSO instead of drug are shown at the top. Colors represent the log_2_ fold change of the EC_50_ of each cell line for a drug relative to the parental cell line’s EC_50_ for that same drug. Data represent the mean of three biological replicates. *, P ≤ 0.05, **, P ≤ 0.01, ***, P ≤ 0.001 (two-sample t-tests with Bonferroni correction).

We observed extensive collateral changes in response to resistance to each of the four drugs we studied here (Fig. 4A–D, Supplementary Table S1). All collateral effects were in the form of resistance. All increases in sensitivity we observed (to statins and the surviving inhibitor YM155) were determined to be artifacts of the culturing system, as a similar fraction of DMSO control cell lines also exhibited increased sensitivity to these drugs, suggesting that the culturing system can exert collateral effects on drug sensitivity.

### Collateral effects of classical chemotherapy resistance are heterogeneous

We observed notable heterogeneity in the collateral drug responses we measured. For example, the paclitaxel-resistant cell lines exhibited a wide range of sensitivities to Actinomycin D (Fig. 4C). To formally assess this heterogeneity, we performed unsupervised hierarchical clustering on the log_2_ fold-changes in sensitivity to the drugs studied (Fig. 4A–D). In the case of doxorubicin and paclitaxel resistance, hierarchical clustering demarcates two clusters of resistant cell lines with distinct collateral drug sensitivity signatures (Fig. 4A,C). This clustering methodology did not clearly distinguish any clusters of vincristine- or cisplatin-resistant cell lines. However, for each drug assayed, we are still able to observe a range of changes in EC_50_ values. This suggests that isogenic cells can respond to the same evolutionary pressure in heterogeneous ways, resulting in diverse phenotypes of the resulting resistant cells.

### Phenotype-based clusters of paclitaxel resistant cell lines are not associated with transcriptomic-based clusters of the same cell lines

**Figure 5:**
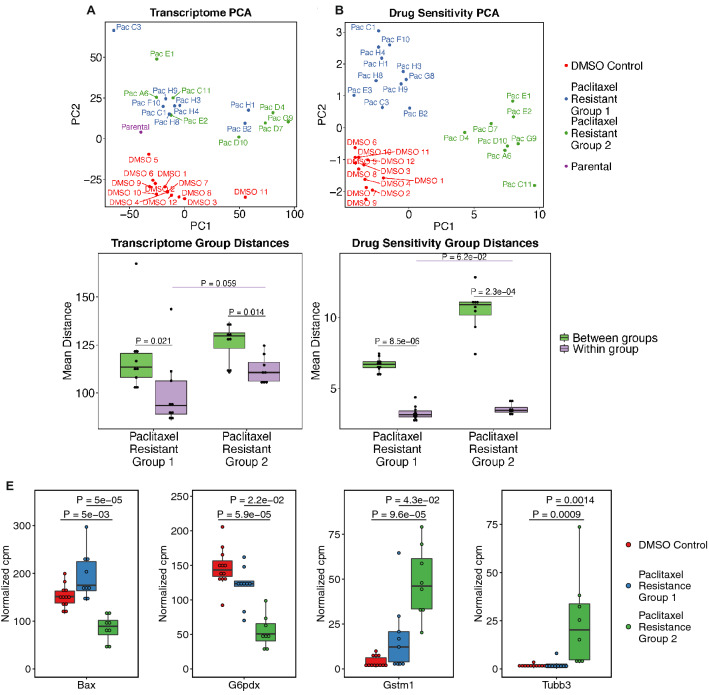
Phenotype-based groups of paclitaxel resistant cell lines. **(A)** log of TMM-normalized counts-per-million expression values from RNA-seq and **(B)** PCA plots of log_2_ EC_50_ fold change values for the paclitaxel resistant cell lines, DMSO control lines, and parental line. The blue and green annotated phenotypic groups are independent of the apparent transcriptome groups (Fisher’s Exact Test P = 0.33). Mean Euclidian distance separating cell lines between and within the phenotype-based paclitaxel resistance groups in **(C)** transcriptome space, and in **(D)** drug resistance space. FDR-corrected P-values are reported for two-sample Wilcoxon tests. **(E)** TMM-normalized counts for genes with a significant Kruskal–Wallis test and significant post-hoc Dunn’s test (Supplementary Fig. S2) are shown. FDR-corrected P-values are shown for post-hoc Dunn’s test of the indicated comparisons.

To study the cause of the observed phenotypic clustering of resistant cell lines, we performed RNA-seq on the paclitaxel resistant cell lines, the DMSO control cell lines, and the parental cell line. Two of the paclitaxel resistant cell lines did not pass QC metrics so 17 of the paclitaxel resistant lines were sequenced. PCA of the transcriptomic data from these cell lines clearly separated the DMSO control lines from the paclitaxel resistant lines, and also revealed two groups within the paclitaxel resistant lines (Fig. 5A). PCA of the phenotypic drug sensitivity data from these cell lines recapitulated the differences between the DMSO control vs. paclitaxel resistant cell lines, as well as the two groups of paclitaxel resistant cell lines we previously observed with hierarchical clustering (Fig. 5B). Paclitaxel Resistance Group 1 exhibited a range of collateral effects, while Paclitaxel Resistance Group 2 became collaterally resistant to most of the drugs we assayed. Surprisingly, the phenotypic-based and transcriptomic-based groups within the paclitaxel resistant lines are independent of each other (Fig. 5A,B). Despite this, in transcriptomic space, cell lines within phenotypic-based groups are significantly closer to other cell lines within their group than to cell lines in the other group (Fig. 5C,D).

### Gene expression sheds light on different resistance mechanisms between phenotype-based groups of paclitaxel resistant cell lines

After observing two phenotype-based groups of paclitaxel resistant cell lines, we were interested in understanding transcriptome-based differences between these groups. However, these groups are interspersed with each other in transcriptome space, so global differential gene expression analysis revealed only two significantly expressed genes—Bst1 and Dglucy both show increased expression in Paclitaxel Resistance Group 1 (Figs. 5, Supplementary Fig. S1A–C & Supplementary Table S2). We next performed gene set enrichment analysis on the genes that are differentially expressed between each group of cell lines. The top gene sets for each comparison were immune related, including interferon alpha and gamma response, TNFα signaling, and inflammatory response (Supplementary Fig. S1D–F).

To further assess additional potential differences in resistance mechanisms between these two groups, we compared expression levels of genes related to established mechanisms of paclitaxel resistance in these two groups as well as the DMSO control lines (Supplementary Table S3). Of the 17 genes with a significant difference in gene expression between these three groups of cell lines, only four, Bax, G6pdx, Gstm1, and Tubb3 showed a significant difference in expression specifically between the two paclitaxel resistance groups (Fig. 5E, Supplementary Fig. S2, & Supplementary Table S4).

We next probed known mechanisms of resistance to paclitaxel by measuring intracellular levels of Abcb1a, Abcb1b, Bcl-xL, and MCP1 via qPCR, and quantifying levels of IL-6 secreted into media. We found that Abcb1a and MCP1 are not expressed in our cell lines, and that Bcl-xL is expressed at a low level in the DMSO control lines as well as both paclitaxel resistance groups. On the other hand, Abcb1b is more highly expressed in both paclitaxel resistance groups than the DMSO control cell lines. There is not a significant difference in Abcb1b levels between the two groups, confirming our observation from transcriptomic data. Finally, levels of IL-6 in media were lower than the limit of detection of our ELISA kit (5 pg/mL) (Supplementary Fig. S3A).

### Paclitaxel resistance group 2 cell lines are collaterally sensitive to verapamil, but paclitaxel resistance is attenuated equally by verapamil co-treatment in both groups

**Figure 6:**
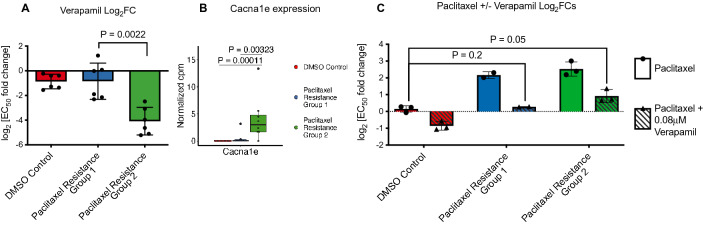
Paclitaxel resistance group 2 cell lines are collaterally sensitive to verapamil, and all paclitaxel resistant lines efflux paclitaxel. **(A)** Log_2_FC of verapamil EC_50_s, relative to the parental cell line. P-value is shown for Mann–Whitney test comparing paclitaxel resistance group 1 and group 2. **(B)** TMM-normalized counts for Cacna1e. FDR-corrected P-values are shown for post-hoc Dunn’s test of the indicated comparisons. FDR correction was performed across all genes with a significant Kruskal–Wallis test and significant post-hoc Dunn’s test including those in Fig. 5E. See Supplementary Table S4 for all genes tested. **(C)** Log_2_FC of paclitaxel EC_50_s, relative to the parental cell line with and without addition of 0.08 μM verapamil. P-values are shown for one-sided Mann–Whitney tests comparing paclitaxel resistance group 1 and paclitaxel resistance group 2 to DMSO control. Paclitaxel EC_50_s with verapamil treatment are not significantly different between paclitaxel resistance group 1 and group 2 (Wilcoxon test p-value = 0.8).

To functionally assess known mechanisms of paclitaxel resistance in our paclitaxel resistant cell lines, we set out to perform paclitaxel dose response curves in the presence and absence of compounds known to modulate pathways previously implicated in paclitaxel resistance. The mechanisms of resistance that we investigated in this manner are: drug efflux via Abcb1 (with verapamil), regulation of apoptosis via Bcl2 (with venetoclax), and glutathione levels (with buthionine sulfoximine). We first performed dose response curves with these compounds alone to identify the highest non-toxic dose to use in the combination treatment. Unexpectedly, we found that Paclitaxel Resistance Group 2 cell lines are significantly more sensitive to verapamil as a single agent than Paclitaxel Resistance Group 1 cell lines. In other words, Paclitaxel Resistance Group 2 cell lines are collaterally sensitive to verapamil (Fig. 6A; Supplementary Fig. S3B,C).

In addition to blocking Abcb1-mediated drug efflux, verapamil is also a calcium channel blocker. Therefore, we tested the hypothesis that calcium flux or signaling may differ between the two groups of paclitaxel resistant cell lines. We compared expression of calcium channels and calcium-dependent kinases between the paclitaxel resistant groups. We found that the calcium channel Cacna1e is more highly expressed in Paclitaxel Resistance Group 2 (Fig. 6B, Supplementary Fig. S2, Supplementary Tables S3 and S4).

Finally, to assess the role of Abcb1 drug efflux, Bcl2 signaling, and glutathione levels on paclitaxel resistance, we next performed paclitaxel dose response curves in the presence or absence of the highest non-toxic dose of the three compounds that inhibit these mechanisms of resistance. Treatment with 0.5 μM venetoclax or 10 μM buthionine sulfoximine did not affect paclitaxel sensitivity in any of our cell lines (Supplementary Fig. S3D,E). Treatment with 0.08 μM verapamil increased paclitaxel sensitivity of all the cell lines, however despite the differential collateral sensitivity to this agent between Paclitaxel Resistance Group 1 vs. group 2, both groups of cell lines were equally sensitized to paclitaxel by verapamil treatment. Verapamil treatment sensitized Paclitaxel Resistance Group 1 cell lines to the level of the DMSO control cell lines. However, Paclitaxel Resistance Group 2 remained significantly more resistant to paclitaxel than the DMSO control lines in the presence of verapamil (Fig. 6C).

## Discussion

We have established a method to reliably generate cell lines resistant to classical chemotherapies. We leveraged this technique to survey phenotypic responses collateral to acquiring classical chemotherapy resistance. Unexpectedly, we found that there was marked heterogeneity in collateral responses across cell lines exposed to the same evolutionary pressures. In fact, we observed that about half of the cell lines that acquired resistance to paclitaxel also acquired a collateral sensitivity to verapamil. This heterogeneity in collateral response across the resistant cell lines suggests the existence of many possible cellular responses to the evolutionary pressure of chemotherapy. This hypothesis is supported by recent work showing that multiple mechanisms of resistance to a drug lead to stochastic collateral effects of resistance to that drug^21^.

The drugs we used in our screen have known resistance mechanisms that can be engineered in the lab such as topoisomerase II mutation or decreased expression causing doxorubicin resistance, and mutations in α- and β-tubulin causing vincristine resistance^9,22,23^. However, after studying sensitivity to other compounds in our screen, in many cases, resistance did not appear to be based on these known mechanisms. For example, many doxorubicin and cisplatin resistant cell lines did not exhibit collateral resistance to 5-FU, a known Abcb1 drug efflux pump substrate^24^. Additionally, doxorubicin resistant cells were never collaterally sensitive to the topoisomerase I poison topotecan, as would be expected if resistance resulted from mutation or decreased expression of topoisomerase II^23^. Similarly, vincristine or paclitaxel resistance was not coincident with collateral sensitivity to the other drug, a result which would have been expected if resistance resulted from mutations in α- or β-tubulin. These results call into question the clinical relevance of the known lab-based mechanisms of classical chemotherapy—if lab-based evolution of resistance does not result from the known mechanisms of resistance, does patient-based evolution of resistance result from those mechanisms? In fact, review of the literature reveals that the relevance of known resistance mechanisms is unclear in many cases, motivating further work to understand the nuances of clinically relevant collateral effects of chemotherapy resistance^25–27^.

The paucity of differentially expressed genes between the phenotype-based groups of cell lines resistant to paclitaxel suggests that most of the alterations acquired during resistance evolution are passengers and unrelated to drug sensitivity. This is supported by our observations that the phenotypic-based and transcriptomic-based groups are not associated with each other. While the phenotypic-based groups do loosely cluster in transcriptomic space, it is clear that many of the alterations acquired are not associated with phenotypic response to other chemotherapies. This has important implications for study of tumor heterogeneity—transcriptomic profiling cannot be used as a proxy for phenotypic profiling because the relevant transcriptomic changes are obscured in a global analysis by unrelated changes caused by adaptation to the drug. In particular, certain approaches such as the connectivity map which seek to infer functional aspects of biology based on a transcriptional state may be misled by the myriad of unrelated variants^28^. Of course, transcriptomic analysis is also hindered by its descriptive nature—it is neither a mechanistic nor functional assessment of cellular behavior.

Combination dosing with verapamil revealed that paclitaxel resistance was largely mediated by Abcb1 drug efflux. Despite this finding, about half of the paclitaxel resistant cell lines (Paclitaxel Resistance Group 1) did not exhibit the broad cross-resistance to other chemotherapeutics expected in cell lines with higher expression of Abcb1. Specifically, cell lines in Paclitaxel Resistance Group 1 had either no or only moderate decreases in sensitivity to Abcb1 substrates including etoposide, doxorubicin, vincristine, 5-FU, and cisplatin, and increased sensitivity to the Abcb1 substrate actinomycin D. Interestingly, verapamil did not fully ameliorate paclitaxel resistance in the 2nd group of paclitaxel resistant cell lines. This group of cell lines (Paclitaxel Resistance Group 2) has increased expression of class III β-tubulin, an alteration that has been widely implicated in paclitaxel resistance both in the lab and in the clinic^29–32^. Interestingly, there are some reports that overexpression of class III β-tubulin can cause resistance to other tubulin binding agents and DNA-damaging agents, potentially explaining the difference in collateral drug sensitivity between the two groups of paclitaxel resistant cell lines^33^.

We also observed that this same group of paclitaxel resistant cell lines (group 2) is collaterally sensitive to verapamil. We suspect that this collateral sensitivity is not due to drug efflux per se, because both paclitaxel resistance groups have similar levels of Abcb1 expression and similar effects of verapamil co-treatment with paclitaxel. While determination of the mechanism of this collateral sensitivity is beyond the scope of this study, we have several hypotheses that could explain these observations. The first is that the cell lines in Paclitaxel Resistance Group 2 may be creating an endogenous toxic compound that is normally effluxed by Abcb1. The second is that verapamil has been shown to induce apoptosis by increasing glutathione efflux via MRP-1^34,35^. Although these cell lines do not have different levels of MRP-1 (Supplementary Fig. S3F), nor do they have a differential response to inhibition of glutathione synthesis, they do have higher expression of the glutathione transferase Gstm1. These cells may have altered glutathione flux that causes the collateral sensitivity to verapamil. Finally, since verapamil alters intracellular calcium levels by blocking calcium channels, alterations in calcium flux or signaling may result in verapamil sensitivity. Intriguingly, one common side effect of paclitaxel treatment is peripheral neuropathy which may result from reduced calcium signaling^36^. We did observe higher expression of one calcium channel, Cacna1e, in cell lines with collateral sensitivity to verapamil, however this observation cannot confirm or reject this hypothesis. Further work is needed to test each of these hypotheses and determine the mechanism of this collateral sensitivity.

Examination of the changes in drug sensitivity across our DMSO control cell lines revealed several curious effects of our culturing system. First, we observed heterogeneity in sensitivity to several drugs including statins, YM155, sorafenib, 5-FU, chlorambucil, and topotecan. We also observed heterogeneity in gene expression among the DMSO control cell lines, although differences between control cell lines were not concordant in transcriptomic and phenotypic space, echoing what we observed when comparing the paclitaxel resistant cell lines. Specifically, the DMSO control lines only shared nearest neighbors in drug sensitivity and transcriptome PCA space in two out of 12 cases (Fig. S3).

We observed that some aspect of the growth conditions during the evolution of resistance, such as the extended culturing time, or culturing in the presence of DMSO caused increased sensitivity to two statins and to YM155 in many of the control cell lines. The mechanism behind the control cell lines’ ‘collateral’ sensitivities to the statins and YM155 is unknown. Recently there has been evidence in the literature that statins may be effective as cancer therapies; however, these results need to be carefully considered if culturing techniques can affect cell lines’ statin sensitivity^37–40^.

After removing artifact drugs for which the DMSO control cell lines exhibited changes in sensitivity, we observed many collateral resistances in our resistant cell lines, but only one collateral sensitivity in eight out of 112 cell lines. Intriguingly, this result supports a recently revived theory termed independent action which posits that drug combinations are effective not because of synergy or other interaction between drugs, but rather because one drug will be effective for each individual cell or tumor. Under this paradigm, drug combinations are effective simply because multiple drugs represent multiple ‘shots on goal’ for an individual in a population with heterogeneous and difficult-to-predict drug sensitivities^41,42^. Current combination regimens are effective largely because the drugs within them act independently of each other, and drug interactions like collateral sensitivity are rare.

The fact that collateral sensitivity to classical chemotherapies is rare even in the single agent setting tested here underlines the reality that our understanding of the short- and long-term effects of these drugs is lacking. In contrast, resistance to targeted therapies is much better understood, and in fact targeted therapies induce collateral sensitivities. However, in the clinic, poor response to 1st-line treatment with classical chemotherapy is predictive of failure of 2nd-line therapies. This observation predicts the result we observed but is complicated by the fact that most patients are treated with more than one agent. Perhaps the early use of classical chemotherapies in treatment is problematic and should instead be a last resort. Instead, to extend patient survival, frontline therapy should consist of combinations of targeted therapies which have more predictable resistance mechanisms and can result in treatable collateral sensitivities.

The sparsity of collateral sensitivities to classical chemotherapies is surprising, as collateral sensitivity has been widely documented in response to antibiotic resistance^3,43–45^, as well as to targeted chemotherapy resistance^6,46^. While antibiotics and targeted therapies are thought to interact relatively specifically with their targets, classical chemotherapies are thought to be more promiscuous and thus have a wider distribution of binding affinities for entities within the cell^47–49^. Our results lead us to hypothesize that collateral sensitivities result from strong evolutionary pressure on one or a small number of specific targets within the cell. In these cases, resistance can result from on-target mutations or target bypass, both of which can produce novel and exploitable sensitivities^46,50–52^. In contrast, the diffuse evolutionary pressure exerted by a compound with many binding partners would not be addressed by any single mutation or bypass mechanism as many cellular processes are affected. In this case, resistance may instead result from a more general state change, for example, to an anti-apoptotic state which would not be vulnerable to inhibition of any specific node within the cell. This paradigm leads to our observed result—rare collateral sensitivities to classical chemotherapies, while others have observed collateral sensitivities to antibiotics and targeted therapies.

**Figure 7:**
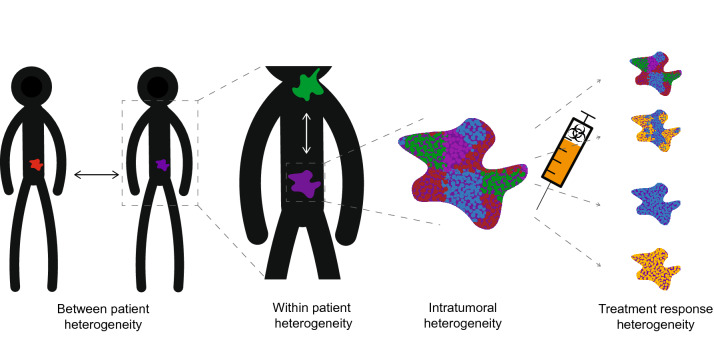
Schematic of a new dimension of tumor heterogeneity. Scientists and physicians have previously investigated heterogeneity between tumors in different patients, heterogeneity between different tumors in the same patient, and heterogeneity within the same tumor. Heterogeneity in tumor response to treatment also needs to be investigated.

Our results, showing the sparsity of collateral sensitivities to classical chemotherapy resistance, motivates further work to better understand mechanisms of action and resistance to these agents. Furthermore, efforts are needed to understand which combinations of classical chemotherapies work well together in order to rationally design novel combination regimens. This work is complicated by our observation of heterogeneity in phenotypic response to acquisition of classical chemotherapy resistance which is not explained by differences in gene expression. The discussion of heterogeneity in cancer has previously focused on inter-patient heterogeneity, heterogeneity between metastases within a patient, and intra-tumor heterogeneity. We have uncovered a new dimension of heterogeneity which needs to be further explored—heterogeneity in response and resistance to chemotherapy (Fig. 7).

## Materials and methods

### Cell culture and chemicals

Murine Eµ-Myc; p19^ARF−/−^ *-*B-cell lymphomas^16^ (a gift from Dr. Charles Sherr, St. Jude) were cultured in B-cell medium *(45%* DMEM, *45%* IMDM, 10% FBS, supplemented with 2 mM l-glutamine and 5 μM β-mercaptoethanol). All cell lines were routinely tested for mycoplasma contamination using MycoAlert (Lonza). All drugs were obtained from LC Laboratories, Sigma-Aldrich, Calbiochem, or Tocris Biosciences. All drugs were dissolved in DMSO except cisplatin, which was dissolved in 0.9% saline.

### Creation of resistant cell lines

10,000 cells were seeded into each well of 96-well plates (Falcon), then treated with a dose of a chemotherapeutic to kill 85% of cells after three days. After treatment, viability was assessed via PI staining and FACS. Cells were spun out of drug and allowed to recover in drug-free media for 4 days. During recovery, cells were split as individual wells’ media color started to change. On day 4, all wells were split to approximately 10,000 cells per well and the removed cells were frozen at −80 °C in PCR plates. The cycle was repeated for 12–18 weeks.

### Cell viability assay

Cells were seeded in cell-culture treated 384-well plates (Falcon), then cells were treated as indicated. Cell viability was measured after 72 h using resazurin sodium salt (Sigma). Resazurin was used at 0.008 mg/mL and plates were analyzed 6 h after addition. Fluorescence was measured using the Tecan M200 Pro at an excitation of 550 nm and emission of 600 nm.

### Calculation of EC_50_s

The following analysis was performed in Matlab: median fluorescence of wells without cells was subtracted from all wells on the same plate. Viability was then normalized to cell growth by dividing all corrected fluorescence values by the median corrected fluorescence of wells containing untreated cells. Viability data was then fit to the following four-parameter hill curve:

1 $$viability= {A}_{inf}+ \frac{{A}_{0}- {A}_{inf}}{1+{\left(\frac{[drug]}{EC50}\right)}^{n}}$$

where A_inf_ is the viability at high doses of the drug, and A_0_ is the viability with low or no drug. Sensitivity metrics were generated for each replicate individually, which were then used for further statistical analyses.

### RNA-sequencing and differential gene expression analysis

Total RNA was prepared from the paclitaxel resistant cell lines, the DMSO control cell lines, and the parental cell line using the RNeasy Mini Kit (Qiagen), per the manufacturer’s protocol, and included the on-column DNAse step (Qiagen).

RNA samples were quantified and quality assessed using an Advanced Analytical Fragment Analyzer. The initial steps were performed on a Tecan EVO150.10 ng of total RNA was used for library preparation. 3’DGE-custom primers 3V6NEXT-bmc#1-15 were added to a final concentration of 1 µM. (5'-/5Biosg/ACACTCTTTCCCTACACGACGCTCTTCCGATCT[BC_6_]N_10_T_30_VN-3' where 5Biosg = 5’ biotin, [BC6] = 6 bp barcode specific to each sample/well, N10 = Unique Molecular Identifiers, Integrated DNA technologies), to generate two subpools of 15 samples each.

After addition of the oligonucleotides, Maxima H Minus RT was added per manufacturer’s recommendations with Template-Switching oligo 5V6NEXT (10 µM, [5V6NEXT : 5’-iCiGiCACACTCTTTCCCTACACGACGCrGrGrG-3’ where iC: iso-dC, iG: iso-dG, rG: RNA G ]) followed by incubation at 42 °C for 90 min and inactivation at 80 °C for 10 min.

Following the template switching reaction, cDNA from 15 wells containing unique well identifiers were pooled together and cleaned using RNA Ampure beads at 1.0×. cDNA was eluted with 17 µl of water followed by digestion with Exonuclease I at 37 °C for 30 min, and inactivated at 80 °C for 20 min.

Second strand synthesis and PCR amplification was done by adding the Advantage 2 Polymerase Mix (Clontech) and the SINGV6 primer (10 pmol, Integrated DNA Technologies 5’-/5Biosg/ACACTCTTTCCCTACACGACGC-3’) directly to the exonuclease reaction. 8 cycles of PCR were performed followed by clean up using regular SPRI beads at 0.6×, and eluted with 20 µl of EB. Successful amplification of cDNA was confirmed using the Fragment Analyzer^53,54^.

Illumina libraries were then produced using standard Nextera tagmentation substituting P5NEXTPT5-bmc primer (25 μM, Integrated DNA Technologies, (5’-AATGATACGGCGACCACCGAGATCTACACTCTTTCCCTACACGACGCTCTTCCG*A*T*C*T*-3’ where * = phosphorothioate bonds) in place of the normal N500 primer.

Final libraries were cleaned using SPRI beads at 0.7× and quantified using the Fragment Analyzer and qPCR before being loaded for paired-end sequencing using the Illumina NextSeq500 in paired-end mode (26/50 nt reads).

Quality control was done with FastQC version 0.11.4^55^. Single-end reads were aligned to the GRCm38 mm10 assembly with gencode M25 annotation using STAR version 2.5.1b^56^. Gene counts from STAR were used to perform differential gene expression analysis with R version 3.6 using edgeR version 3.32.0^57,58^. Gene expression differences at the total gene level were considered significant at an FDR of < 0.01.

### Gene set enrichment analysis

GSEA was performed on the described differentially expressed genes using clusterProfiler^59^ using the hallmark gene sets in MSigDB^60^ with an FDR correction and a p-value cutoff of 0.05. enrichplot was used to make the GSEA plots^61^.

### qPCR

RNA was prepared from the indicated cell lines using the Quick-RNA Miniprep Kit (Zymo Research) and reverse transcribed with SuperScript IV Reverse Transcriptase (Thermo Fischer). Real-time PCR reactions were performed using PrimeTime Gene Expression Master Mix (IDT) on a LightCycler 480 II (Roche). Data were analyzed using the ΔΔCT method, and relative mRNA levels were normalized to Pgk1 levels. Primers used were IDT Predesigned Taqman Gene Expression Arrays for for Bcl-xL (Mm00437783_m1), Mcp-1 (Mm00441242_m1), Abcb1a (Mm00440761_m1), Abcb1b (Mm00440736_m1), and Pgk1 (Mm.PT.39a.22214854).

### ELISA assays

The indicated cell lines were seeded in 6-well tissue culture plates for 24H. The media from those plates was centrifuged to remove cells, supernatant collected, and stored at −80 °C until measuring of IL-6 levels with a kit from Thermo Fisher (88-7064-88).

### Statistical analysis

Statistics were performed using GraphPad Prism 5 (GraphPad Software Inc) and R version 3.6. All error bars represent standard error of the mean.

## Supplementary Information

Supplementary Figure S1.

Supplementary Figure S2.

Supplementary Figure S3.

Supplementary Figure S4.

Supplementary Information.

Supplementary Table S1.

Supplementary Table S2.

Supplementary Table S3.

Supplementary Table S4.

## Data Availability

The datasets generated during and/or analyzed during the current study are available in the GEO repository under accession number GSE166425. All data needed to evaluate the conclusions in the paper are present in the manuscript.
