# Supplementary material for: Collateral responses to classical cytotoxic chemotherapies are heterogeneous and sensitivities are sparse
Source: Sci Rep. 2022 Mar 31;12:5453. doi: 10.1038/s41598-022-09319-1 (PMC8971507; doi:10.1038/s41598-022-09319-1)
Supplement: Supplementary file 5 — Supplementary Information. [file 41598_2022_9319_MOESM5_ESM.docx]

Supplementary Information

Title: Collateral Responses to Classical Cytotoxic Chemotherapies are Heterogeneous and Sensitivities are Sparse

**Authors:** Simona Dalin^1,2^, Beatrice Grauman-Boss^1,2^, Douglas A. Lauffenburger^1,3,*^, Michael T. Hemann^1,2,*^

**Affiliations:**

^1^Department of Biology, Massachusetts Institute of Technology, Cambridge, MA.

^2^Koch Institute for Integrative Cancer Research, Massachusetts Institute of Technology, Cambridge, MA.

^3^Department of Biological Engineering, Massachusetts Institute of Technology, Cambridge, MA.

* To whom correspondence should be addressed:

Michael T. Hemann, Koch Institute, Massachusetts Institute of Technology, 77 Massachusetts Ave., Building 76 Rm 361, Cambridge, MA 02139. Phone: (617) 324-1964; Fax: (617) 258-6172; E-mail: [hemann@mit.edu](mailto:hemann@mit.edu).

Douglas A. Lauffenburger, Department of Biological Engineering, Massachusetts Institute of Technology, Room: 16-343, Cambridge MA 02139. Phone: (617) 252-1629; Fax: (617) 258-0204; E-mail: lauffen@mit.edu.

**Including:**

- Supplementary figure and table legends
- Supplementary references

**Supplemental Figures and Legends:**

**Fig. S1** Gene expression differences between phenotype-based paclitaxel resistant cell line groups. **(A-C)** Volcano plots of differential gene expression between, **(A)** DMSO control and Paclitaxel Resistant Group1, **(B)** DMSO control and Paclitaxel Resistant Group 2, and **(C)** Paclitaxel Resistant Group 1 and Paclitaxel Resistant Group 2. Genes highlighted in red have greater than 1.5-fold change in gene expression, and FDR > 0.01. **(D-F)** Enrichment of hallmark gene sets in genes differentially expressed between **(D)** DMSO control and Paclitaxel Resistant Group1, **(E)** DMSO control and Paclitaxel Resistant Group 2, and **(F)** Paclitaxel Resistant Group 1 and Paclitaxel Resistant Group 2. NES = normalized enrichment score.

**Fig. S2** Differences in gene expression for paclitaxel resistance related genes between phenotype-based paclitaxel resistant cell line groups. TMM-normalized counts for genes with a significant Kruskal-Wallis test are shown.

**Fig. S3** Buthionine Sulfoximine and Venetoclax do not affect paclitaxel resistant cell lines **(A)** qPCR measurement of levels of Abcb1a, Abcb1b, Bcl-xL, and Mcp-1 in the DMSO control and paclitaxel resistant cell lines relative to levels in the parental cell line. ND = not detected. **(B-C)** Dose response curves with buthionine sulfoximine (B) and venetoclax (C) in the DMSO control and paclitaxel resistant cell lines. **(D-E)** Log_2_FC of paclitaxel EC_50_s, relative to the parental cell line with and without addition of 10 μM buthionine sulfoximine (D) or 0.5 μM venetoclax (E). **(F)** TMM-normalized counts for Mrp-1 are shown. Result of Mann-Whitney test comparing paclitaxel resistance group 1 to group 2 is shown. NS = not significant.

**Fig. S4** Heterogeneity and phenotype/transcriptome discordance among DMSO control cell lines. **(A)** PCA plots of log_2_ EC_50_ fold change values and **(B)** log of TMM-normalized counts-per-million expression values from RNA-seq for the DMSO control lines.

**Table S1** Log_2_ fold changes and EC_50_ values for each drug and cell line tested.

**Table S2** Global differential gene expression between each paclitaxel resistant group and DMSO control group. For Paclitaxel Resistant Group 1 vs. DMSO control group, Paclitaxel Resistant Group 2 vs. DMSO control group, and Paclitaxel Resistant Group 1 vs. Paclitaxel Resistant Group 2, results of differential gene expression analysis with EdgeR are reported.

**Table S3** Genes proposed to be involved in resistance to paclitaxel, based on a literature review. Gene name, description, proposed paclitaxel resistance mechanism, and P-value, and FDR of Kruskal-Wallis test comparing expression between the two paclitaxel resistant groups are included. See supplemental references for citations. See Supplemental References for references

**Table S4** Dunn Post-Test results of gene expression differences between each paclitaxel resistant group and DMSO control group. For each gene and comparison indicated, the Dunn test Z-score, P-value, and FDR-adjusted P-value is reported.

**Supplemental References**

1. Kavallaris, M. Microtubules and resistance to tubulin-binding agents. *Nat. Rev. Cancer* **10**, 194–204 (2010) PMID:20147901.

2. Chen, Z. *et al.* Mammalian drug efflux transporters of the ATP binding cassette (ABC) family in multidrug resistance: A review of the past decade. *Cancer Lett.* **370**, 153–164 (2016) PMID:26499806.

3. Gupta, N., Gupta, P. & Srivastava, S. K. Penfluridol overcomes paclitaxel resistance in metastatic breast cancer. *Sci. Rep.* **9**, 1–14 (2019) PMID:30911062.

4. Wang, Y. *et al.* Autocrine production of interleukin-6 confers cisplatin and paclitaxel resistance in ovarian cancer cells. *Cancer Lett.* **295**, 110–123 (2010) PMID:20236757.

5. Kutuk, O. & Letai, A. Alteration of the mitochondrial apoptotic pathway is key to acquired paclitaxel resistance and can be reversed by ABT-737. *Cancer Res.* **68**, 7985–7994 (2008) PMID:18829556.

6. Feng, Q. *et al.* Targeting G6PD reverses paclitaxel resistance in ovarian cancer by suppressing GSTP1. *Biochem. Pharmacol.* **178**, 114092 (2020) PMID:32535103.

7. Murphy, M., Hinman, A. & Levine, A. J. Wild-type p53 negatively regulates the expression of a microtubule- associated protein. *Genes Dev.* **10**, 2971–2980 (1996) PMID:8956998.
